# Accessing targeted therapies for cancer: self and collective advocacy alongside and beyond mainstream cancer charities

**DOI:** 10.1080/14636778.2020.1868986

**Published:** 2021-01-17

**Authors:** Anne Kerr, Choon Key Chekar, Julia Swallow, Emily Ross, Sarah Cunningham-Burley

**Affiliations:** a University of Glasgow, Glasgow, UK; b Faculty of Health and Medicine, Lancaster University, Lancaster UK; c University of Edinburgh, Edinburgh, UK

**Keywords:** precision oncology, access, patients, solidarities, advocacy

## Abstract

As precision oncology has evolved, patients and their families have become more involved in efforts to access these treatments via fundraising and campaigning that take place outside of the larger cancer charities. In this paper, we explore the solidarities, networks, and emotional work of the UK-based access advocates, drawing on the stories of nine advocates, which included interviews and content analyses of their social media posts and coverage of their case in news, commentary, and fundraising websites. We consider the emotional and knowledge work of building networks that spanned consumerist and activist agendas, forged individual and collective goals, and orientations toward the public, private, and third sectors as part of securing support and access. Through these various practices, the actors we have studied cultivated personal advantage and solidarities with other patients and advocates, and in so doing engaged in self and collective advocacy alongside and beyond mainstream cancer charities.

## 1. Introduction

Personalized medicine has become an important goal of contemporary medicine, particularly via tailored or targeted therapies of cancers that are designed to act on specific molecular targets to slow or arrest cancer growth (Ross *et al*. 2004). Hailed as the ultimate form of individualized medicine, targeted therapies for cancer are strongly associated with personal narratives of hope and survival. Industry and government, together with cancer charities and healthcare providers across the private and public sectors, have come together to develop and promote access to these new opportunities through a range of trials, studies, and novel treatments. Although filled with promise, targeted therapies are experimental, expensive, and complex, making widespread access difficult across both insurance-based and state-funded healthcare systems. Pharmaceutical companies have, therefore, sought to increase awareness of their potential benefits among patients and doctors and accelerate regulatory approval of targeted therapies and companion diagnostics. For example, in 2019 Bayer reportedly planned to spend $70M to increase patient and physician awareness of testing for rare mutations and encourage regulatory approval of more tests as a means of enabling the use of more targeted therapies such as Vitrakvi. This targets cancers with a rare genetic mutation found in less than 1% of solid tumors. This effort includes a public awareness campaign, “Test your cancer”, that encourages patients to ask their doctors about genomic testing to enable early access to appropriate therapies (Steenhuysen and Burger 2019; see also https://global.testyourcancer.com/global). Crowdfunding for medicines has also grown in recent years, including for targeted therapies, promoting what Berliner and Kenworth have called “forms of individualized charity” (2017, 233). As Lee and Lehdonvirta (2020, no pagination) argue these form an “entrepreneurial safety net: one where protection is not afforded universally or on the basis of need, but on the basis of one’s ability to appeal to the audience.” Through these educational fundraising and media campaigns around access to treatments, contemporary priorities of individual empowerment, deservingness, and the promise of cure are reinforced.

Nevertheless, concerns abound in the medical and social science literatures about the hype, limited evidence of effectiveness, and inappropriate pricing of targeted therapies for cancer (Davis 2015; Maughan 2017; Wiersma *et al*. 2019). Davis’s (2015) study of what she calls “chemotherapeutic expansion”, based on interviews with cancer patient advocates, regulators, specialists, and other stakeholders in the US and EU, suggests that experimental therapies with limited evidence of efficacy proliferate because of permissive regulatory standards, which prioritize the development of these therapies as a means to grow national bioeconomies, exploiting the hopes of desperate patients with advanced disease. At the same time, critics have argued that “access-advocacy” (Mayer 2003) does not present a sufficient challenge to industry practices – it is overly-reliant on and uncritical of industry knowledge and support, focusing instead on the failings of public health systems unable to finance these expensive treatments. This lack of scrutiny of industry is intensified by the media and charities amplifying messages of hope around cancer therapies and cure:

Publication bias, distorted scientific reporting, promotional material, and stories of “miracle” drugs percolate against a background discourse of “science at a crossroads” and “new eras”. (Davis 2015, 212)

This renders patients vulnerable to exploitation in and through a media captured by the corporate interests of the pharmaceutical industry. Gabe *et al*.’s (2012) study of debates around access to Herceptin in New Zealand found that, although they are not monolithic and operate in “loose alliances”, patient and consumer groups tend not to counter, but rather align with industry interests, with the media playing various roles of providing information and constructing a “debate” around access often deploying a news frame of “desperate” patients. In a recent study of the largest US patient advocacy organizations, of which around 30% were cancer-related, over 80% received financial support from the biotechnology, medical devices, or pharmaceutical industry (McCoy, Carniol, and Chockley 2017).

Hope is an important conduit through which these alliances are sustained and replicated. Scholarship, on the political economy of hope invested in cancer therapeutics (Del Vecchio Good *et al*. 1990; Jain 2013), has demonstrated how hope enfolds the individual obligation to be positive and active in the fight against cancer. As Ehlers and Krupar (2014) have written, “hope is now seen not as a social and collective expression of national belonging and welfare, but instead as something potentially embodied in one’s own biological material and facilitated by biotech advancements and corporations” (2014, 396). Oncologists and patients become responsible for cultivating and maintaining hope, including via a “fighting spirit” where together they will battle against barriers to access, side effects, low mood and mutations, enacting “the biopolitical imperative to enhance and optimize life” (Ehlers and Krupar 2014, 407) via the latest “cutting-edge” treatments and trials.

Digital media platforms are increasingly important means by which patients are active and engaged in these individual battles (Lupton 2014; see also Vicari and Cappai 2016). Cancer patients are active participants in these platforms as they gather and share knowledge around living with cancer, taking part in research and accessing information, tests, treatments, and care (Ziebland and Wyke 2012). Western-centric notions of survivorship, enablement, self-responsibility, and cheerfulness are common, notably in breast cancer fora (Orgad 2005). As Petersen and colleagues argue, there is also growing emphasis on patients working with science and business on profile – and fund-raising online (2019). These activities are amplified and monetized by traditional media and digital media which “align with a consumer-driven model of digital patient activism” (Petersen, Schermuly, and Anderson 2019, 489).

Patient campaigns and alliances can nevertheless also involve more critical engagement with the interests of industry and neoliberal imperatives of positivity and empowerment. Barbot (2006), in a study of AIDS associations in France, suggests that their knowledge work can take various forms that might involve a more questioning approach, including orientation to science as a learner, experimental subject, or citizen scientist. Here patients are part of new arrangements between the state and the market. Rabeharisoa, Moreira, and Akrich (2014) have argued that this kind of “evidence-based activism” reimagines illness and its treatment as part of new forms of social relationships which are often condition-focused, knowledge-laden, and transformative. These activities reframe what is at stake, destabilizing and reworking existing understandings of conditions and associated problems, working across and developing new kinds of expertise in the process. Although this can align with corporate or state-based interests, it can also challenge established institutions and practices. Breast cancer activists’ focus on the environmental causes of breast cancer is a good example of critical engagement that challenges industry to go beyond the search for cure (McCormick, Brown, and Zavestoski 2003). O’Donovan (2007) has also demonstrated the cautious relationship of patient organizations engaging with the pharmaceutical industry. As Vicari and Cappai (2016) note, in a study of rare disease patients online, fluid connections and activities among patients can also go beyond traditional or “top-down” arrangements for patient groups with defined leaders and institutional structures, with platforms like Facebook and Twitter being important means through which patients shared knowledge and developed political agendas together.

In this article, we contribute to the study of patient advocacy, focusing on advocates’ efforts to access targeted therapies for cancer, which largely take place outside of established cancer charities and patient organizations. We explore how these activities involved knowledge and emotional work that aligned with and challenged individualized quests for self-fulfillment and cure, industry and state-based healthcare institutions. Drawing on 9 advocate’s stories from a multi-sited ethnographic study of personalized cancer medicine, we explore the work and networks involved in the quest for tailored treatments and the kinds of capital and relationships that are formed in the process. We focus on stories of individual determination and deservingness as well as solidarities with other patients in accounts and engagements online. We argue that these cases suggest that patients and their supporters are engaging in not only self advocacy but also collective action which combines knowledge and emotional work to promote and challenge the NHS, charitable sector and, to a lesser extent, the pharmaceutical industry.

## 2. The study

The UK provides fertile territory for a study of individual and collective ambitions and values when it comes to personalized medicine given its growing private healthcare sector and continuing state-based provision via the National Health Services. Not all targeted cancer therapies are free-at-the-point-of-use in the NHS, either because the drugs have not been approved for use by the regulator (e.g. the National Institute for Clinical Excellence in England), or because the local health body (e.g. Clinical Commissioning Group (CCG) in England) has decided not to fund treatments for particular patients based on the details of their case. Typically, these drugs are only made available to a sub-group of patients with specific cancer types and treatment histories, via trials, private medicine or when there is sufficient evidence of benefit to justify their use as part of NHS care. The regulatory process that determines access via the NHS involves considerable dialogue and revision as evidence and interpretation unfold – trial data can be incomplete, local health bodies are subject to challenges and appeals from patients and their advocates, as are pharmaceutical companies accused of predatory pricing by the regulator. Charities and other advocacy and campaigning organizations acting on behalf of patients are also involved in these debates and discussions, alongside the media, which regularly profile patients seeking treatments not approved by their local NHS. In recent years, the phenomenon of crowdfunding has also grown online and patients and their supporters have begun to raise funds for targeted therapies in this way. The BBC recently reported that £7 m was raised for cancer treatments via crowdfunding sites like JustGiving and GoFundMe in the three years up to 2017, although the proportion of funds, raised for targeted as opposed to other treatments including alternative medicine, is not known.^1^

Our study of cancer patient, carer, and advocate efforts to access targeted therapies not freely available on the NHS developed from a wider ethnographic study of cancer patienthood and genomic medicine, which was largely based around case studies of NHS trials, feasibility studies, and standard care involving molecular profiling, Whole Genome Sequencing and/or targeted therapies (see Kerr *et al*. 2021). We identified instances of access advocacy for targeted therapies for cancer in interviews with some patients, carers, and professionals and we conducted online searches of UK press coverage, social media, charity websites, and patient forums. Following this process of scoping and review, we identified five sets of activities used by patients/carers to access drugs: self-funding via insurance/private treatment; crowdfunding; challenges to NHS/NICE-restricted access; advocacy for pricing reform; and, advocacy for access to trials/more biobanking research. Using snowballing sampling, we went on to conduct further interviews with advocates for access to targeted therapies who were prominent campaigners on social media, focusing on the first four of these activities. We focused on these activities because they were smaller scale, typically patient or carer driven, and they tended to sit outside the main charitable or research institutions. This contrasted with advocacy for access to trials (e.g. actforcancer.org.uk, set up in the wake of the death of the prominent Labor politician Tessa Jowell, from brain cancer), which was typically more embedded in larger charitable and research institutions so less patient or carer led and therefore difficult to access first person patient and carer accounts. The bulk of our analysis is drawn from online materials, supplemented by interviews with a small sample of key advocates. Accessing interviewees was difficult due to the ill-health of cancer patients and because the activities of carers and advocates were small scale, limiting the pool of potential interviewees. The details of our data are set out below:

**Table:** 

	Interviews	Online data sources
(i) Self-funders: Jill – bowel cancer patient with private insurance, prescribed Avastin (not available on NHS) [Jill’s relative also set up a crowdfunding page for “alternative therapies” for Jill]; Douglas – bowel cancer patient self-funding Avastin	We interviewed Jill and Douglas – both were paying for treatments privately or via insurance schemes while also advocating for wider access to targeted therapies.	We analyzed Jill’s and Douglas’s blogs and Twitter posts. We followed their links to patient forum discussions, charitable websites, and campaigns and analyzed this material too [with permission to quote]
(ii) Crowdfunding: Gillian – ovarian cancer patient, fundraising for targeted therapies that she may need in future and alternative therapies; Sarah – breast cancer patient, fundraising for targeted therapies not available on NHS, e.g. Kadcyla; Claire – breast cancer patient, fundraising for targeted therapies not available on the NHS e.g. Kadcyla; Tom – bowel cancer patient, fundraising for targeted therapies not available on NHS e.g. Avastin; Rachel – ovarian cancer patient, fundraising for targeted treatment not available on the NHS	We conducted 2 interviews with Gillian and Sarah.	We analyzed Gillian’s and Sarah’s and three other crowdfunding fundraising pages (Claire, Tom, Rachel) and their blogs/ Twitter/Facebook/Instagram posts. We also analyzed media coverage of these patients’ stories [with permission to quote from Gillian and Sarah]
(iii) Appeals to NHS commissioners/challenges to NICE: Sasha	We interviewed Sasha, a carer who became a campaigner and set up a foundation for cancer patients seeking the challenge NHS decisions to refuse access to targeted therapies, focusing on NHS Trust commissioning decisions	We analyzed the foundation website and Sasha’s social media posts [with permission to quote]
(iv) Advocacy/pricing reform: Mark	We interviewed Mark, an activist advocate who set up a small campaigning foundation to advocate for patients seeking access to targeted therapies (for cancer and other diseases) focusing mainly on challenging pharmaceutical companies’ pricing policy and NICE decision-making	We analyzed the advocacy foundation website, including features on patient ambassadors, associated social media posts, academic reports linked to the campaign, media coverage, and political party policy making [with permission to quote]
*Totals 9 stories*	*6 interviewees*	*4 blogs (Jill, Douglas, Gillian, Sarah); 5 fundraising pages (Gillian, Sarah, Claire, Tom, Rachel); 2 foundation websites (Sasha, Mark); 5 Twitter accounts (Jill, Douglas, Gillian, Sasha, Mark); 2 Facebook accounts (Gillian, Claire); 1 Instagram account (Gillian); Patient forum discussions (Jill, Douglas); Newspaper and TV coverage (Douglas, Gillian, Sarah, Claire, Tom, Rachel, Mark); Charity website (Jill, Douglas, Gillian, Sarah, Claire, Rachel); Various academic reports and political party policy documents (connected to Mark’s foundation)*

In the analysis that follows we explore the knowledge and emotional work of forging networks and support, including orientating to institutions (NHS, charities) and industry (pharma), and framings of individual and collective problems and solutions, particularly in relation to entrepreneurialism, hope, consumption, and solidarity. We have given our participants’ pseudonyms and all data were collected with appropriate University ethical approval.^2^ We utilized situational analysis as a theory/methods package to capture and analyze the situations, commitments, and controversies in which advocates operated, attending to complexities of discourses, agencies, and structures (Clarke, Friese, and Washburn 2016).

## 3. Analysis

### 3.1. Building networks

Being a cancer patient is a full time job,” says Spurrier. “If you want some normality, like non-patients have, then you have to be extremely organised and knowledgeable … Patients invest everything in their treatment and survival, much of which is unrecorded and unevaluated, but we are not nearly as good at managing and optimising the impact of it on our lives as engaged, chronic patients sometimes are. (Wagstaff 2017)

It is widely acknowledged that a cancer diagnosis brings with it a lot of work to try to stay well or get better by gathering knowledge, managing schedules, relationships, and emotions (Hubbard, Kidd, and Kearney 2010; Martin and Finn 2011; Jain 2013; Gibson, Lee, and Crabb 2015). For some patients, accessing targeted therapies not otherwise available as part of public-funded healthcare can become a vital part of this work – researching options, sharing experiences with others in similar situations, advocating for policy, institutional or industry changes, and fund-raising for treatments.

In our study, we found that tending to wide-ranging networks of people engaged with or affected by similar types of cancer (depending on its origins in the body, stage, and genetic sub-type) or seeking the same or other similar targeted therapies was fundamental to gathering support, influence, and opportunities for this kind of campaigning and advocacy. On-line networking was particularly important, not just for those actively seeking donations on crowdfunding platforms toward their own treatment, but for others seeking to raise their profile in order to recruit allies, clients, or cases to campaign around. For access advocates, this included developing an array of online activities such as blogs, social media accounts on Twitter, Instagram, and Facebook pages, petitions, and photoshoots as well as coverage by their local newspapers, national press, charity websites and short films, and in some cases television coverage on news channels and documentaries. As Van Dijck and Poell (2013) have noted, these processes of cross-syndication entwine social and mass media logics. Attending to and extending workplace, patient, family, and friendship networks was part of sourcing support and donations. This could include connections to journalists to gain coverage about their situation, often via local papers taking up their stories. For example, one crowdfunding patient, Tom, had a relative who was a journalist and he also got coverage of his situation in the national press because of his existing profile as a worker in the cultural industries. Coverage of awards, such as Campaigner of the Year or Inspirational Woman of the Year from charities, in local media outlets was a feature of this work too.

This networking spanned professional and patient contacts. After one of her parents died from cancer, Sasha told us how she curated a range of contacts in oncology as part of the campaigning work she developed, and sought out clients to leverage influence:

It’s really important for me to keep that contact, because that, for one, keeps me knowledgeable about what the actual process is …  what they’re going through, and another gives me a really powerful patient group …  that are behind me, that …  obviously need the change.

A notable feature of these networks was how they spanned different kinds of advocacy and fund-raising practices, sharing knowledge, experiences and support, and campaigning with self-funders and crowd-funders, people who were fortunate to have access to insurance and others who were not, and encompassing consumerist and activist agendas. Sasha and Mark, for example, were working on different tactics to improve access to a range of targeted therapies, with Sasha developing networks with wealthy philanthropists and private physicians as well as patients and their families to advocate for patient consumers and challenge the NHS, whereas Mark was networking with other activists and campaigners in the UK labor movement, as well as patients and their families to challenge pharma, some of whom also worked with Sasha.

Ensuring the diversity and flexibility of networks was connected to the need to build support, followers, and “likes” online. For advocates with a professional background in an adjunct field, which included public relations professionals, writers, public speakers, or teachers, this could involve providing press packs, which included a note of follower numbers on the main social media platforms. But extending networks was important for others too. For crowdfunders this was a matter of generating more donations. For more entrepreneurial advocates who had developed a livelihood connected to their cancer advocacy – sourcing funds and clients for their foundation, selling their books or art, teaching, or coaching – it could also generate more income. However, even for the advocates who were not directly involved in raising funds, networks were important as a means of developing social and emotional support, recognition, and self-worth. As Gillian wrote in her blog:

…  it’s friends old and new rallying around me, sending me gifts, and keeping me amused and entertained throughout that I have been, and continue to be, most overwhelmed by. Cancer is all-consuming, relentless and exhausting, but worst of all it’s lonely. That is why I am eternally grateful to you all for sticking with me and keeping me afloat. It goes some way to counteract the truly darkest of days.

Experiencing and performing validation was important in and of itself for those seeking funds for treatment too, so much so that their identity as a cancer patient and an accomplished writer, sports person, or campaigner, came to the fore in some of their networking rather than their efforts to raise funds or advocate for tailored treatments. In some media or blog discussions on their situation their crowdfunding activities, the details of their cancer or tailored drugs sought were not mentioned at all. Rather the focus was on their accomplishments or campaigning work, sometimes, though not always, in alliance with charities – for example, in one media article about Tom, his flourishing creativity was the focus; and in Gillian’s blog her many awards, invitations, and features in the press were celebrated as “honours” or “highlights”.

These overlapping, flexible, and multiple networks (Vicari and Cappai 2016) spanned a range of political and economic positions and agendas, and in so doing combined individual and collective kinds of advocacy involving considerable emotional and knowledge work, as we now go on to consider in more depth.

### 3.2. Individual and collective advocacy

As we have already discussed, advocates’ efforts to access targeted therapies were part of a wide repertoire of entrepreneurial activities, such as working as educators, artists, writers, or campaigners with cancer and treatment access motivating and becoming a focal point for their outputs. For example, following on from her experiences of trying to access a particular targeted therapy for a parent with cancer, Sasha developed a full-time campaigning role, advocating for patients’ refused access to other kinds of targeted therapies by NHS commissioners. Developing a network of patients unable to access treatments was also important for other self-funders and advocates in our study like Mark and Jill, who gave coverage and support to these cases to extend their network and amplify their campaign. This involved a range of knowledge and emotional work. Sasha stressed the way in which her campaigning work built upon and developed her public relations skills and knowledge of NHS Commissioning, inventing a new kind of role that the NHS should embed in their services – drawing on her passion and drive to improve access to present her as a trouble-shooter who could improve a failing service.

Crowdfunders were entrepreneurial in sourcing “individualized charity” (Berliner and Kenworthy 2017, 233), including through developing support networks, profiling, and organization of a range of fundraising activities for their cause. This was often done indirectly – fundraising sites were sometimes hosted by relatives or included posts that raised the profile of relatives and friends raising funds on their behalf, for example through different kinds of charity concerts and sporting pursuits, amplifying the positive emotions of making a difference by fundraising and expressing gratitude without directly asking for donations to their own healthcare.

However, these activities also went beyond individual advocacy, cultivating solidarities with a purpose beyond individual goals of accessing targeted therapies, encompassing advocacy, advice, and care for other patients, with some crowdfunders featuring as ambassadors in Mark’s foundation’s campaigns, for example. Private funders were similarly engaged in acts of solidarity with other patients and carers, as access to targeted therapies became a collective rather than a straightforwardly individual problem. Their focus was on the entitlements of cancer patients to access the right drugs for their cancer rather than on particular drugs or even cancer patients with particular molecular subtypes of cancer. Advocates also used their social media platforms to profile and lend support to other advocates and campaigns, including the work of charities on wider awareness-raising campaigns focusing on screening and prevention, for example.

The entrepreneurial spirit was entwined with a collective ethos in these various individual and collective pursuits, spanning efforts to achieve transformation for self and others, marked by emotions of hope and also concern for others, courage, and determination. For example, in a discussion about what motivated her to become a writer, teacher, and advocate for her and other patients, Gillian wrote in her blog:

I was not prepared to be a statistic. I was not prepared to die. I am going to be the change.

Being part of a community seeking change involved encouraging donations and profiling of others seeking targeted therapies and giving advice about new trials or treatments abroad that might be useful and making an emotional connection with others. For example, in one comment on one of the fundraising sites, we analyzed an anonymous donor wrote:

I know my donation is small but I hope it helps a little – I have also started a go fund me for my husband as he is struggling through cancer right now. One thing we found very helpful was looking at personal diagnostics. You should check our Craig Venter’s Health Nucleus. That’s what we’re trying to raise money for. Hang tight and stay strong.

Arguably this advice magnified individual goals of personalization, but it also offers reciprocal care and support in a collective sense.

Obligations to keep fighting cancer were also tied to frequent expressions of gratitude or demonstrations of deservingness, often linked to a sense of obligation to stay positive, well, or alive for children or spouses. This included accounts of a sense of reciprocity to donors and other supporters, as well as “giving back” to the NHS or cancer community more generally. We found both an individual and collective ethos in accounts and videos that access advocates posted about their experience or reactions to donations or support from others. In one video, Tom’s tearful partner tells us about her gratitude to donors and tells poignant stories about people who have donated after their own cancer experiences, reflecting that this is because people are responding to Tom’s goodness and his contributions to other people’s lives through his creative work. Here individual attributes and deservingness and collective values of compassion and empathy for others were mutually reinforced.

These stories of connection emphasized comfort as well as shared resolve. For example, Claire, another crowdfunder, made requests for donations on her blog and expressed hopes that her health would improve as a result of targeted therapies becoming available to her, but she also wrote about how important it was to share her story with and meet other women affected by cancer, for example on a course she recently attended, as it offered hope for the future. Access advocates built a profile and a platform online which went beyond sharing knowledge, sourcing funds or advocating for individuals to be able to get targeted therapies on the NHS, to encompass the emotional work of caring about the lives of cancer patients more generally, be that patients with the same cancer (e.g. being positive about “living life” with other “stage 4 bowelies”), or challenging the UK government to reassure “cancer friends” about the supply of cancer drugs post-Brexit.

As we’ve discussed, hope was important throughout these cases, but it sat alongside other emotional work around individual and collective deservingness, modesty, and vulnerability. For example, in one post Claire appealed to her deservingness by giving an account of her hopes and doubts. She wrote about how her clinicians were happy that she has new tailored treatment options on the NHS, but she was doubtful because she had become used to having her hopes dashed and could not believe this will be any different. Claire vowed to continue researching her cancer and raising funds, but acknowledged it’s not so urgent now she has other treatment options. She ended her post with a joke about a haircare gift being a sign of her optimism. Elsewhere on her blog Claire focused more on collective agendas for access and care, joining together with other kinds of access campaigns, including those run by charities. She noted the sense of purpose that these activities gave her and emphasizes the reciprocities involved – asking for help with campaigning to support her because of her own track record in supporting others.

Douglas also expressed modest hopes for his own future in his posts about his experiences of cancer where he includes discussion about self-funding:

I’ve been self funding to the tune of a little over £10,000 for 6 treatments! Despite being available in other countries, in the UK [the drug’s] effectiveness …  is viewed as inconclusive. In my position (and luckily I can afford it for now) I felt compelled to give it a go.

…  I received the result of my latest scan and it was a mixed bag! The tumours …  responded well to treatment and have reduced nicely. But unfortunately I’ve got some tough little bastards in my lungs and they’ve actually grown. The long and the short of it is that I’ll be back on chemo …  in 3 weeks time. …  It does seem that perhaps the tide of good news is beginning to turn. While I’m realistic, don’t bet on me not beating the odds!

Here Douglas, like Claire, calibrated his hopes and tried to be realistic, given his prognosis. Disappointments were moderated by humor and maintaining hope about “beating the odds”. We can also find other kinds of appeals to collective benefits and hopes on Douglas’s social media alongside these more individual stories. For example, Douglas reflected in one blog post about what is good about living with cancer, including getting together with friends to fundraise for a charity. He continued:

We all have different ways of coping, but the disease can be quite isolating, and I really believe that there are amazing benefits to be had through sharing with others going through similar experiences. Many have extraordinary stories and all have been through tough times, but there is a real sense of “we’re all in this together.”

Access advocacy could also generate as well as counter difficult emotions. In the case of crowdfunders in particular, cultivating interest and support by posting intimate details of their daily life and illness experiences or displays of emotion online could be wearing, as could frequent attendance at fundraising events organized by supporters. For some this generated anxiety about meeting the expectations of donors or other patients who were also fundraising e.g. donating to others or offering advice about how to fundraise. Here the obligations of supporting and building networks came to the fore. Engagement and advocacy for access more generally could also generate distress. For example, in a media feature about one of the crowdfunders in our study, the writer noted Tom’s passion and determination to see improvements in cancer care promised by personalization and his difficulties with managing the anger and distress he felt about the impact of the current political situation on cancer services and health care more generally.

### 3.3. NHS, charities, and pharma

As the discussion thus far has illustrated, self and collective advocacy involves a plethora of knowledge and emotional work, including in relation to the NHS, charities, and the pharmaceutical industry, and it is to this that we now turn to consider in more depth.

We found that access advocates were especially engaged with the role of the NHS in providing targeted therapies. Even for patients accessing treatment privately, the NHS was where their treatment had begun, and accessing therapies privately often took place on NHS premises or in combination with other kinds of care freely provided by the NHS. Considerable emphasis was placed on the need for the NHS to deliver these treatments at no cost, across advocates’ activities and accounts, whatever their approach to funding treatment. Sometimes this involved challenging commissioning bodies, as in Sasha’s case, where the NHS was cast as a faceless and outdated bureaucracy, as in the tweet below:

Last night I received desperate letters of a man fighting bureaucracy to stay alive. 25 yrs old, no “standard” cancer treatment left … His peers in the US are receiving immunotherapy – he can’t understand why he can’t have the same and a chance to live. I’ll fight for him now … 

Emotive comparisons with the treatments available to patients in the USA were used by Sasha to advance the case for better access in the UK. At other times advocates focused on drawing patients together with political activists to campaign against the US-based pharmaceutical companies and pressuring NICE to enable the NHS to deliver the best available treatments, as in Mark’s case. Other patients worked on campaigns with political parties and charitable bodies that included but went beyond efforts to access particular targeted treatments. For example, one crowd-funder, Claire, worked with a breast cancer charity to campaign for everyone to get the treatments they needed and also featured in their campaign about how the NHS is failing women with breast cancer because of delays in diagnosis.

Yet advocates also praised and expressed support and devotion to the NHS across their digital content and interview accounts. Mark worked with political parties to advocate for new modes of pharmaceutical pricing to protect the NHS from profiteering. Claire met with politicians and worked with another access-advocacy group where her story was featured on their website and she positioned her as a supporter of the NHS, criticizing government under-funding. Others commented on media reporting about waiting lists and political party policies on the NHS. Tom, for example, criticized the government for trying to privatize the NHS and the effect this would have on those living in poverty and called for support for the NHS. The NHS was also positioned as struggling to provide by other crowdfunders, such as Rachel, who sought to pressurize the NHS to provide treatments at the same time as she expressed gratitude to its staff for the care she has received.

In an interview with another crowdfunder, Sarah, we can see how orientation NHS could be a matter of careful reflection about tensions around privilege and solidarity:

I’m fortunate enough to be in a position where I am able to write up my story in an articulate …  logical manner …  as clearly as I can so that people know exactly what I’m fundraising for and why I’m doing it. …  not everyone is a position to be able to do that … . that is really sad because … we’re discriminating against people who need …  the help as much as anyone else. …  So we’re coming back to …  poor people being worse off …  it’s all becoming like a two-tier system, …  and it …  should be …  healthcare for all. That’s the premise of the NHS and …  it’s dividing people again.

As this quote illustrates, even for self-funders, solidarity with patients and the collective spirit of the NHS was important.

Personal links to charities were also important for the advocates we studied. These also spanned self and collective advocacy. These links were built through advocates’ re-posting social media material from charities, acting as ambassadors for campaigns, appearing in the media or in short films for the charity as well as attending charity events and meetings.

As we’ve already discussed, patients like Claire worked with particular charities to advocate for prevention and access to treatments, including features about their case in short films and articles on charity blogs, sometimes also featuring in news media. While these activities did not raise funds for drugs directly, they did create reach and likeability – vital to extending networks of potential donors. However, engaging with charities, like engaging with other patients and carers, was not simply instrumental; instead, it was part of cultivating a sense of solidarity and compassion. This was part of being an advocate beyond access to medicines for oneself, as Gillian noted:

… so I support …  the two ovarian cancer charities and raise money for them just to try and get the awareness around that. But …  I don’t know …  [it doesn’t get more attention] it’s not …  sexy enough [as compared to breast cancer where there is more campaigning and funding]

Gillian’s campaigning included appearing in teal colored underwear as part of ovarian cancer awareness week, one part of a busy cancer-based working life, writing, teaching, and healing others. Note, however, that elsewhere Gillian reflected on her blog about the need to center on her and reduce the amount of campaigning and support she offered to others as her illness advanced.

Crowdfunding advocates also signposted donors and supporters to particular charities on their fundraising sites. This included indicating how their funds would be used to support charities if they were not used for treatment costs, and updates about charity events attended. Crowdfunders also got involved in fundraising for charities when their illness had abated, for example Rachel posted a link to a sponsored sporting event she was participating in as part of fundraising for a local hospice on her crowdfunding page.

Just as with tensions around criticizing and supporting the NHS, at time advocates also become involved in criticizing charities, as one self-funder, Jill, explained. Jill experienced charity sites as problematic from the start of her cancer:

I was getting very, very frustrated that …  the general ethos was ‘be positive and don’t worry about life, eat lots of cake and be happy

Here Jill found the ethos of positivity and hope cultivated by charities jarred with her own drive to improve care. She intervened, asking other patients what tests they’ve had and discussing diet, but was asked by moderators to avoid giving dietary advice, as this should be the province of bona fide experts. Although this decreased her engagement with charities Jill nevertheless approached a charity to try to join forced around advocacy for drug access:

With the [drug] issue …  – and that was when I wrote my blog post – …  it was such a contentious issue for me that I contacted [a charity] because I thought …  if I go to NICE and challenge them, they’re just gonna bat me away, I’m one person. But if I can do it through …  the cancer charity, perhaps they’ve got …  a bit more voice. And I said to them, “Look, I would be prepared to head up the campaign, do all the work for it, alongside other patients because there are a few patients who are quite willing to do it, but we need your name and your guidance. …  to know exactly who we approach.” And they just said to me they’re not interested …  in pursuing it because they’ve tried before; NICE have said no, it’s very unlikely they’re ever gonna change their mind on it, so there’s no point in challenging it anymore.

This experience led Jill to set up her own closed Facebook group to support and advocate for other patients who seeking tailored treatments, including through crowdfunding, although she maintained a link with the charity and their CEO later joined in the Facebook group.

There was less engagement with and critical reflection about the pharmaceutical industry across the accounts we analyzed, although there were also clear instances of criticism from some actors, notably Mark, Tom, and Claire. The focus of this criticism was on profiteering, as Mark noted, saying that his campaign work is:

very much focused on the responsibility of first and foremost, the pharmaceutical companies, for …  charging a price which is justifiable and affordable, and ethical.

However, for campaigners like Claire, alliances were also built with pharma, for example when she spoke at and event sponsored by industry to share her story and build awareness of the needs of patients like her. Sasha also told us she had “made a conscious decision not to get involved with the pharmaceutical pricing” in her campaign work and to remain focused on the NHS. Although she articulated support for other campaigns that were critical of excessive pharmaceutical pricing, she also went on to praise the good work of the industry, expressing concern that they could be “unfairly demonized”, and noting that she has “met some really lovely people within the industry that are doing amazing things” to facilitate patient access. Other patients were also ambivalent or largely silent about criticism of pharmaceutical companies. For example, Gillian, a blogger with an extensive repertoire of connections to charities and cancer writing and support work, positions pharmaceuticals as “Western medicine”, which she uses alongside traditional therapies, seeing this as one part of her activities as a warrior against cancer, and did not engage with questions of the ethics of pricing or pressures on the NHS.

## 4. Discussion and conclusion

The range and variety of these accounts of the NHS, charities, and pharmaceutical industry, above, demonstrate that these kinds of access advocates combine self and collective advocacy in complex and diverse ways. They cannot be sorted into categories of passive dupes, naive consumers, or antagonists. Although there was not a great deal of critical engagement with pharmaceutical industry practices or the efficacy of the kinds of tailored treatments being advocated, and a tendency to focus on the problems of the NHS (Mayer 2003), this was complicated by other kinds of critical engagement with industry and NHS funding which shifted the discussion to wider political and economic processes. Consumer-oriented logics of entitlement to access tailored therapies to extend individual survival and benefit sat within a wider terrain of hopefulness for equity for other and future patients and services, where solidarity came to the fore. Here we see a version of Rabeharisoa, Moreira, and Akrich’s (2014) “evidence-based activism” not only reimagining illness and its treatment as part of condition-focused patient collectives, but also tentatively reimagining the relationship between patients, charities, the health service, and industry.

As targeted therapies for cancer evolve, so too do the ways in which patients and their supporters advocate for access for themselves and others. Traversing analogue and digital worlds, creating new networks and livelihoods, working through their orientations toward the state, the third sector and the market, participants in our research were engaged in self and collective advocacy around patient access to targeted therapies and improvements in care more generally. Connected by shared but nonetheless at times contradictory and inconsistent hopes and aspirations, these advocates were neither simply individual consumers of particular treatments or collectivized critics of pharma or the NHS, but instead were involved in building common causes and shared agendas with an array of patients, supporters, and advocates alongside efforts to access particular treatments for their cancers. Hopeful attention to the possibilities of their own therapeutic salvation interspersed their accounts and activities, but it would be wrong to cast this as naïve or misguided, as their hopes were shot through with critical self-reflection and concern for others. Developing their networks as a way to enhance and extend their life also involved sometimes uncomfortable and difficult interactions and exchanges with social and other media providers as details of intimate experiences are made public in exchange for the possibility of more support. These networks brought together people with similar cancers or seeking similar drugs and also included connections to patients and supporters with different illness experiences, backgrounds, and political orientations in common cause, as they worked to support and encourage each other in their quest for tailored therapies. Personal goals for tailored treatments that would extend life were articulated through and in relation to a more collective set of aspirations and goals, where solidarity and equity were key shared concerns.

Social scientists have framed drug development and uptake as a process of “pharmaceuticalization” – “[t]he transformation of human conditions, capacities or capabilities into opportunities for pharmaceutical intervention” (Williams, Martin, and Gabe 2011, 711), highlighting the lack of critical engagement of patient advocate and their supporters. But our study suggests that there is more diversity, skepticism, and critical self-reflection in the work of access advocates than this framing implies. Hope was a key aspect of their activities, but this was not uncritical and could involve critique of charities, industry, and the NHS. Hope also formed one part of a much wider plethora of emotional work that took place alongside the knowledge work of network building, individual and collective activism, and engagement with institutions and industry.

Our analysis suggests the need to further examine the confluence of knowledge and emotional work that make up patient and their supporters’ individual and collective quest for access to targeted therapies as personalized medicine advances. We need to consider further the intersections of digital and analogue worlds and profiles, and the wider terrain of collective and individual advocacy this involves. We have begun to sketch out some of the orientations to the NHS, charities, and pharma of advocates, but further work is needed to explore how these orientations evolve alongside the market, NHS, and charity services in the coming years.

## Notes

1 https://www.bbc.co.uk/news/health-45509942 Accessed 10/9/19.

2 Ethics reference number: AREA 15–108 (University).

## References

[CIT0001] Barbot, Janine. 2006. “How to Build an “Active” Patient? The Work of AIDS Associations in France.” *Social Science & Medicine* 62 (3): 538–551. 16046248 10.1016/j.socscimed.2005.06.025

[CIT0002] Berliner, L. S., and N. J. Kenworthy. 2017. “Producing a Worthy Illness: Personal Crowdfunding Amidst Financial Crisis.” *Social Science & Medicine* 187: 233–242. 28274601 10.1016/j.socscimed.2017.02.008

[CIT0003] Clarke, A. E., C. Friese, and R. Washburn. 2016. *Situational Analysis in Practice: Mapping Research with Grounded Theory*. London: SAGE.

[CIT0004] Davis, C. 2015. “Drugs, Cancer and End-of-Life Care: A Case Study of Pharmaceuticalization?” *Social Science & Medicine* 131: 207–214. 25533871 10.1016/j.socscimed.2014.12.007 PMC4376382

[CIT0005] Del Vecchio Good, M. J., B. J. Good, C. Schaffer, and S. E. Lind. 1990. “American Oncology and the Discourse on Hope.” *Culture, Medicine and Psychiatry* 14 (1): 59–79. 10.1007/BF00046704 2340733

[CIT0001a] Ehlers, N., and S. Krupar. 2014. “Hope Logics: Biomedicine, Affective Conventions of Cancer, and the Governing of Biocitizenry.” *Configurations* 22 (3): 385–413.

[CIT0006] Gabe, J., K. Chamberlain, P. Norris, K. Dew, H. Madden, and D. Hodgetts. 2012. “The Debate about the Funding of Herceptin: A Case Study of ‘Countervailing Powers’.” *Social Science & Medicine* 75: 2353–2361. 23036988 10.1016/j.socscimed.2012.09.009

[CIT0008] Gibson, A. F., C. Lee, and S. Crabb. 2015. “‘Take Ownership of Your Condition’: Australian Women’s Health and Risk Talk in Relation to their Experiences of Breast Cancer.” *Health Risk & Society* 17 (2): 132–148.

[CIT0009] Hubbard, G., L. Kidd, and N. Kearney. 2010. “Disrupted Lives and Threats to Identity: The Experiences of People with Colorectal Cancer within the First Year Following Diagnosis.” *Health: An Interdisciplinary Journal for the Social Study of Health, Illness and Medicine* 14 (2): 131–146. 10.1177/1363459309353294 20164162

[CIT0010] Jain, S. L. 2013. *Malignant: How Cancer Becomes Us*. Oakland: University of California Press.

[CIT0011] Kerr, A., C. K. Chekar, J. Swallow, E. Ross, and S. Cunningham-Burley. 2021. *Personalised Cancer Medicine: Future Crafting in the Genomic Era*. Manchester: Manchester University Press. 33555770

[CIT0012] Lee, S., and Lehdonvirta. 2020. “New Digital Safety Net or Just More ‘Friendfunding’? Institutional Analysis of Medical Crowdfunding in the United States.” *OSF Preprints*, April 6. 10.31219/osf.io/9kecq

[CIT0013] Lupton, D. 2014. “The Commodification of Patient Opinion: The Digital Patient Experience Economy in the Age of Big Data.” *Sociology of Health & Illness* 36 (6): 856–869. 24443847 10.1111/1467-9566.12109

[CIT0015] Martin, G. P., and R. Finn. 2011. “Patients as Team Members: Opportunities, Challenges and Paradoxes of Including Patients in Multi-Professional Healthcare Teams.” *Sociology of Health & Illness* 33 (7): 1050–1065. 21668454 10.1111/j.1467-9566.2011.01356.x

[CIT0016] Maughan, T. 2017. “The Promise and the Hype of ‘Personalised Medicine’.” *The New Bioethics* 23 (1): 13–20. 28517988 10.1080/20502877.2017.1314886 PMC5448398

[CIT0017] Mayer, M. 2003. “From Access to Evidence: An Advocate’s Journey.” *Journal of Clinical Oncology* 21 (2): 3881–3884. 14551309 10.1200/JCO.2003.07.217

[CIT0018] McCormick, S., P. Brown, and S. Zavestoski. 2003. “The Personal is Scientific, the Scientific is Political: The Public Paradigm of the Environmental Breast Cancer Movement.” *Sociological Forum* 18 (4): 545–576.

[CIT0019] McCoy, M. S., M. Carniol, and K. Chockley. 2017. “Conflicts of Interest for Patient-Advocacy Organizations.” *New England Journal of Medicine* 376 (9): 880–885. 10.1056/NEJMsr1610625 28249131

[CIT0020] O’Donovan, O. 2007. “Corporate Colonization of Health Activism? Irish Health Advocacy Organizations’ Modes of Engagement with Pharmaceutical Corporations.” *International Journal of Health Services* 37 (4): 711–733. 18072317 10.2190/HS.37.4.h

[CIT0021] Orgad, S. 2005. “The Transformative Potential of Online Communication.” *Feminist Media Studies* 5 (2): 141–161.

[CIT0022] Petersen, A., A. C. Schermuly, and A. Anderson. 2019. “The Shifting Politics of Patient Activism: From Bio-Sociality to Bio-Digital Citizenship.” *Health: An Interdisciplinary Journal for the Social Study of Health, Illness and Medicine* 23 (4): 478–494. 10.1177/1363459318815944 30526091

[CIT0023] Rabeharisoa, V., T. Moreira, and M. Akrich. 2014. “Evidence-Based Activism: Patients’, Users’ and Activists’ Groups in Knowledge Society.” *BioSocieties* 9 (2): 111–128.

[CIT0024] Ross, J. S., D. P. Schenkein, R. Pietrusko, M. Rolfe, G. P. Linette, J. Stec, N. E. Stagliano, G. S. Ginsburg, W. F. Symmans, L. Pusztai, and G. N. Hortobagyi. 2004. “Targeted Therapies for Cancer 2004.” *American Journal of Clinical Pathology* 122 (4): 598–609. 10.1309/5CWPU41AFR1VYM3F. 15487459

[CIT0025] Steenhuysen, J., and L. Burger. 2019. “‘Inside Drugmakers’ Strategy to Boost Cancer Medicines with ‘Lazarus Effect’.” 6 September (Reuters). Accessed 10 September 2019. https://uk.news.yahoo.com/inside-drugmakers-strategy-boost-cancer-050524462.html.

[CIT0026] Van Dijck, J., and T. Poell. 2013. “Understanding Social Media Logic.” *Media and Communication* 1 (1): 2–14.

[CIT0027] Vicari, S., and F. Cappai. 2016. “Health Activism and the Logic of Connective Action. A Case Study of Rare Disease Patient Organisations.” *Information, Communication & Society* 19 (11): 1653–1671. 10.1080/1369118X.2016.1154587 PMC4959124 27499676

[CIT0028] Wagstaff, A. 2017. “Does Being a Patient have to be a Full-Time Job?” *Cancerworld*, 2 August, no pagination. https://cancerworld.net/patient-voice/does-being-a-patient-have-to-be-a-full-time-job/.

[CIT0029] Wiersma, M., N. Ghinea, I. Kerridge, and W. Lipworth. 2019. “‘Treat them Into the Grave’: Cancer Physicians’ Attitudes Towards the Use of High-Cost Cancer Medicines at the End of Life.” *Sociology of Health & Illness* 41 (2): 343–359. 30460710 10.1111/1467-9566.12830

[CIT0030] Williams, S. J., P. Martin, and J. Gabe. 2011. “The Pharmaceuticalisation of Society? A Framework for Analysis.” *Sociology of Health and Illness* 33 (5): 710–725. 21371048 10.1111/j.1467-9566.2011.01320.x

[CIT0031] Ziebland, S. U. E., and S. Wyke. 2012. “Health and Illness in a Connected World: How Might Sharing Experiences on the Internet Affect People’s Health?” *Milbank Quarterly* 90 (2): 219–249. 10.1111/j.1468-0009.2012.00662.x PMC3460203 22709387

